# Principles of mitochondrial fusion and fission cycle in neurons

**DOI:** 10.1186/2193-1801-4-S1-L34

**Published:** 2015-06-12

**Authors:** Allen Kaasik

**Affiliations:** University of Tartu, Estonia

**Keywords:** Mitochondrial dynamics, neurons, mitophagy

Mitochondrial fusion-fission dynamics play a crucial role in many important cell processes. These dynamics control mitochondrial morphology, which in turn influences several important mitochondrial properties including mitochondrial bioenergetics and quality control, and they appear to be affected in several neurodegenerative diseases. The molecular machineries behind mitochondrial fusion and fission events are relatively well known. The regulation of fusion and fission events beyond the molecular machinery involved is less clear, fusion and fission are not random occurrences but form a cycle whereby fission typically follows fusion. Mitochondrial fission machinery may somehow sense mitochondrial length and become active when the mitochondrion is oversized and cease when mitochondria are smaller. In contrast, mitochondrial fusion events depend heavily on mitochondrial trafficking. Fusion only takes place when two mitochondria meet and motile mitochondria will be more likely to encounter one another. In cultured cortical neurons, for example, only one in every 14th contact between mitochondria results in fusion. The purpose of this presentation is to provide insight into the complex crosstalk between different processes involved in mitochondrial fusion-fission dynamics and to discuss the potential physiological purpose of mitochondrial fusion and fission.

